# COVID-19 RECOVERY: LESSONS LEARNED AND POLICY ACTIONS FOR THE FUTURE

**DOI:** 10.1093/geroni/igac059.029

**Published:** 2022-12-20

**Authors:** Christine Mueller, Kirsten Corazzini

## Abstract

Focusing on Covid-19 recovery, this symposium will feature a selection research studies that highlight lessons learned that are applicable across the continuum of care for older adults, their families and care partners. The collection of presentations bring perspectives from local to global. Each of the presenters will highlight implications for health and aging policy.

